# Association of albuminuria with incident atrial fibrillation in healthy older adults

**DOI:** 10.1093/ckj/sfaf119

**Published:** 2025-04-17

**Authors:** James B Wetmore, Jonathan C Broder, Amy Brodtmann, Andrew M Tonkin, Robyn L Woods, Anne M Murray, Sharyn M Fitzgerald, Johannes T Neumann, Rory Wolfe, Kevan R Polkinghorne

**Affiliations:** Division of Nephrology, Department of Medicine, Hennepin Healthcare, Minneapolis, MN, USA; Hennepin Healthcare Research Institute, Minneapolis, MN, USA; School of Public Health and Preventive Medicine, Monash University, Melbourne, VIC, Australia; School of Public Health and Preventive Medicine, Monash University, Melbourne, VIC, Australia; Cognitive Health Initiative, Central Clinical School, Monash University, Melbourne, VIC, Australia; School of Public Health and Preventive Medicine, Monash University, Melbourne, VIC, Australia; School of Public Health and Preventive Medicine, Monash University, Melbourne, VIC, Australia; Division of Geriatrics, Department of Medicine, Hennepin Healthcare, Minneapolis, MN, USA; Berman Center for Outcomes and Clinical Research, Hennepin Healthcare Research Institute, Minneapolis, MN, USA; School of Public Health and Preventive Medicine, Monash University, Melbourne, VIC, Australia; School of Public Health and Preventive Medicine, Monash University, Melbourne, VIC, Australia; Department of Cardiology, University Heart and Vascular Center, University Medical Center Hamburg-Eppendorf, Hamburg, Germany; School of Public Health and Preventive Medicine, Monash University, Melbourne, VIC, Australia; School of Public Health and Preventive Medicine, Monash University, Melbourne, VIC, Australia; Department of Nephrology, Monash Medical Centre, Monash Health, Clayton, VIC, Australia; Department of Medicine, Monash University, Clayton, VIC, Australia

**Keywords:** albuminuria, ASPREE, atrial fibrillation, UACR

## Abstract

**Background:**

Development of atrial fibrillation (AF) portends an increased risk of adverse cardiovascular outcomes. Relatively little is known about the longitudinal association between urine albumin excretion and the development of AF, particularly among generally healthy older individuals.

**Methods:**

In a *post hoc* analysis of the ASPirin in Reducing Events in the Elderly (ASPREE) trial, we utilized a marginal structural model to quantify the relationship between annual urine albumin:creatinine ratio (UACR) measurements and new-onset AF. Two approaches were used for handling missing data: one utilized multiple imputation and the other used only cases in whom complete information at baseline was available coupled with last observation carried forward for missing data after baseline.

**Results:**

UACR data were available for 19 114 participants (mean age 75.1 ± 4.5 years, 56.4% female, 91.3% White). The median follow-up was 4.7 years. UACR values were low: mean UACR was 2.1 ± 8.2 mg/mmol at baseline and 2.1 ± 3.3 mg/mmol at year 7. AF developed in 941 individuals. The rate of AF development was 1.11/100 person-years among participants with UACR <3 mg/mmol at baseline and 1.74/100 person-years among participants with UACR ≥3 mg/mmol at baseline. After adjustment for a broad range of factors, the hazard ratio for new-onset AF was 1.16 [95% confidence interval (CI) 1.11–1.21] when multiple imputation was used and 1.15 (95% CI 1.10–1.19) when only cases with complete baseline information were used.

**Conclusions:**

In older individuals who had low levels of albuminuria, doubling of UACR was associated with a 16% increase in the risk of new-onset AF.

## Graphical Abstract

**Graphical Abstract:**
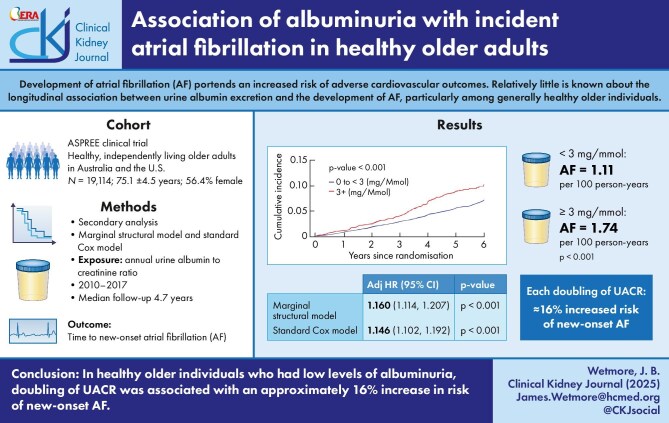

KEY LEARNING POINTS **What was known:**

- Atrial fibrillation (AF), the most common sustained arrhythmia, is a major risk factor for adverse outcomes, including stroke and all-cause mortality.
- Albuminuria, even at very low levels, may be a marker of endothelial dysfunction and associated processes such as inflammation, sympathetic activation and activation of the clotting cascade.
- The level of albuminuria has been shown to be predictive of new-onset (incident) AF.

**This study adds:**

- Healthy older individuals with baseline urinary albumin:creatinine ratio (UACR) levels above the normal range (≥3 mg/mmol) had ≈50% higher unadjusted rates (1.74 versus 1.11 per 100 person-years) of developing new-onset atrial fibrillation compared with those with normal UACR levels.
- When using serial measurements of albuminuria, a doubling of UACR was associated with an approximate 16% increase in the risk of incident AF after adjustment for many other factors.
- The association of albuminuria with AF was similar by strata of age, sex, race/ethnicity, hypertension, body mass index and frailty, although not with diabetes.

**Potential impact:**

- Longitudinal increases in albuminuria among otherwise healthy older individuals appear to portend the risk of new-onset AF, providing a possible rationale for serial measurements of albuminuria in such individuals.
- Employing strategies to reduce albuminuria, such as the use of inhibitors of the renin–angiotensin–aldosterone system, may one day demonstrate utility in reducing the risk of new-onset AF.

## INTRODUCTION

Atrial fibrillation (AF), the most common sustained arrhythmia [1], is a major risk factor for stroke and all-cause mortality [2]. Reduce estimated glomerular filtration rate (eGFR), the most commonly used measure of kidney function, is associated with AF, and as the stage of chronic kidney disease (CKD) worsens, the prevalence of AF increases [3].

Authors of a recent systematic review and meta-analysis identified nearly three dozen studies that quantified the risk of AF associated with eGFR [4], but relatively few that examined the association of albuminuria with incident AF. Although single (baseline) measurements of albuminuria have been associated with the development of AF [3, 5, 6], the implications of longitudinal measurement of albuminuria (i.e. albuminuria measured repeatedly over time) for the development of AF remain incompletely understood, particularly among individuals with very low levels of albuminuria. Albuminuria at low levels—even within the normal range—may be a marker of endothelial dysfunction and associated processes such as inflammation, sympathetic activation and activation of the clotting cascade [7–9] rather than of kidney disease per se. For example, AF is common in older people, but it is not known whether albuminuria is associated with AF in generally healthy older individuals who have had no prior major cardiovascular events. Additionally, previous studies typically utilized only a single assessment of albuminuria at baseline when determining the risk of subsequent AF, a design weakness in view of the variation in albumin excretion that commonly occurs [10, 11].

To better understand how longitudinal (time-varying) measures of albuminuria are associated with the development of AF independent of eGFR in healthy older individuals, we conducted a *post hoc* analysis of the ASPirin in Reducing Events in the Elderly (ASPREE) clinical trial [12, 13]. The trial characteristics of ASPREE make it ideal for the present study: a large well-characterized trial population in which known AF was an exclusion criterion, a long mean duration of follow-up, annual in-person assessments during which AF could be detected and measurements of albuminuria obtained on a yearly basis. We investigated this question using a marginal structural model, an approach that allowed us to control for the potential of time-dependent confounding. We hypothesized that even in a large population of relatively healthy older people with low levels of albuminuria and cardiovascular disease, albuminuria would be associated with new-onset AF.

## MATERIALS AND METHODS

### Study design and population

This study was a secondary analysis of the ASPREE study (2010–2017) [12, 13]. Briefly, ASPREE was a double-blind, placebo-controlled, randomized clinical trial designed to determine whether 100 mg of aspirin, taken as a primary prevention measure, improved disability-free survival in an older, generally healthy population. A total of 19 114 participants were recruited in Australia and the USA. Eligible participants had to be ≥70 years of age and ≥65 years of age for Hispanic and Black participants recruited from the USA. Individuals with a history of AF, prior cardiovascular disease events, dementia, independence-limiting physical disability or other known conditions expected to limit life to <5 years were excluded. Individuals with systolic blood pressure (SBP) ≥180 mmHg or diastolic blood pressure (DBP) ≥105 mmHg were excluded. Participants underwent comprehensive in-person annual study visits that included laboratory sampling (blood and urine) and updates to the medical history.

### Primary exposure

The primary exposure was albuminuria, defined as the urine albumin:creatinine ratio (UACR), ascertained at annual study visits. To better understand the relationship of albuminuria—especially very low levels of urine albumin excretion—with AF, our approach was agnostic as to whether the participant met clinical criteria to be labelled ‘albuminuric’ (e.g. when using a Kidney Disease: Improving Global Outcomes approach). Urine collections could occur in the morning or afternoon, depending on the time of the study visit. Determination of urine albumin and creatinine concentrations were performed in local laboratories using standardized clinical pathology methods. The majority (79%) of UACRs were reported as exact values. As has been described previously [14], the remaining non-missing measures were reported as ‘less than’ a particular value in Australia. In these instances, the reported upper limit of the value was used for analysis. In the USA, the remaining non-missing measures were reported in ranges (<0.11, <3.39, 3.39–33.9 and >33.9 mg/mmol). For these results, the values 0.11, 2.99, 10.7 and 34 mg/mmol were used for analysis, respectively.

### Outcome

The primary outcome for this study was the development of new-onset AF. Ascertainment of AF was conducted by study staff in a standardized fashion using a probabilistic algorithm. Trigger events included new self-reports of AF, new irregular heart rate noted on annual assessments or a new prescription for anticoagulant medications (excluding aspirin), cardiac glycosides or antiarrhythmics. To maximize specificity of the outcome, only individuals with probable (rather than possible) AF were considered to have developed AF. The clinical documents of those with trigger events were reviewed to determine whether it was probable that a new AF diagnosis had been made. Among these reviewed cases, AF was assigned as probable if there was clinical documentation of AF (medical report, electrocardiogram report, admission note) or if the participant had multiple AF reports together with new anticoagulant use, as these cases were highly indicative of AF. Paroxysmal AF that did not result in a diagnosis of AF in the medical record or new use of anticoagulants would not have been labelled as probable AF and therefore would not have been included. Further details have been reported previously [15, 16].

### Covariates

Baseline variables used in the analysis were age, sex, race/ethnicity (Australian White, US White, US Black, US Hispanic, Other), level of education (<12 years, 12–15 years, ≥16 years), living status (living alone versus not), diabetes mellitus and hypertension (no hypertension, i.e. no pharmacologic treatment and SBP <140 mmHg and DBP <90 mmHg; controlled hypertension, i.e. pharmacologic treatment and SBP <140 mmHg and DBP <90 mmHg; uncontrolled hypertension, i.e. SBP ≥140 mmHg or DBP ≥90 mmHg on no pharmacologic treatment; and untreated hypertension, i.e. SBP ≥140 mmHg or DBP ≥90 mmHg on no pharmacologic treatment) as morbidities, dyslipidaemia [use of cholesterol-lowering medications or total cholesterol ≥5.5 mmol/l for Australia and ≥6.2 mmol/l for the USA, or ≥4.1 mmol/l low-density lipoprotein (LDL) cholesterol], irregular heart rate detected at the baseline study visit, measured SBP and DBP, measured heart rate, key laboratory values [high-density lipoprotein (HDL) and non-HDL cholesterol and haemoglobin], current smoking and alcohol use, body mass index (BMI), frailty status (not frail, pre-frail, frail), polypharmacy (defined as the use of more than four prescription medications) [17], use of angiotensin-converting enzyme inhibitors (ACEIs) or angiotensin receptor blockers (ARBs) (due to the effect of these medications on albumin excretion) and treatment arm (aspirin versus placebo). Irregular heart rate detected at the baseline study visit had to be confirmed by the participant's primary care provider as not being AF prior to participant randomization. Haemoglobin was included because anaemia has been associated with the development of AF [18, 19]. Creatinine-based eGFR was calculated using the 2021 Chronic Kidney Disease Epidemiology Collaboration equation [20] and was included as a time-varying covariate in the analysis.

As was done in previous analyses [6], we included two other covariates for time-dependent modelling: acute myocardial infarction and hospitalization for heart failure. These key events were selected because they can increase albuminuria and the risk of (future) AF [21], meaning that they could be time-dependent confounders of the relationship between albuminuria and AF.

### Statistical analysis

Baseline characteristics were summarized by baseline UACR (<3 versus ≥3 mg/mmol), with numerical variables reported as means and standard deviations (SDs) and categorical variables as counts and percentages. UACR mean and SD were presented over the follow-up. The competing-risks Nelson–Aalen cumulative incidence of AF was plotted according to baseline UACR. Rates of AF (per 100 person-years) during follow-up were also presented by baseline UACR.

The association between time-varying UACR and time to AF was analysed using a marginal structural Cox model with truncated weights as the primary approach [22]. Given the aetiological nature of the research question, cause-specific hazards were estimated, with deaths treated as censoring events. UACR was analysed as a continuous variable in a time-updated fashion. Inverse probability weights at each year were calculated to address time-varying confounders (eGFR, acute myocardial infarction and hospitalized heart failure). Linear regression was used to estimate weights based on baseline covariates, time-varying confounders and year, which was modelled as a cubic spline to account for non-linear annual changes in UACR. UACR was transformed for use in the weight-generating model and the outcome model because it followed a skewed distribution; log_2_ was used (as previously in Polkinghorne *et al.* [14]), which enabled the interpretation of model coefficients as the change in risk associated with a doubling of UACR.

To avoid extreme inverse probability weights (and the variance they may induce), weights were stabilized and truncated at the 0.1 and 99.9 percentiles. Balance in time-varying confounders was determined by assessing the correlation between log_2_ UACR and time-varying confounders at each year, where |correlation| < 0.1 was defined as balanced [23]. The outcome model was further adjusted for baseline covariates because weight stabilization balances only time-varying confounders [22]. To counteract the potential bias introduced by truncation, we also modelled using the untruncated weights as a sensitivity analysis. In addition, to evaluate the impact of accounting for time-varying confounders with the inverse probability weights, we performed a standard Cox model as an additional sensitivity analysis.

Several approaches to the handling of missing data were considered. First, we utilized multiple imputation (30 imputations and 10 iterations) using chained equations [24]. Imputation models included time varying and baseline covariates, as well as AF status and the estimated cumulative hazard of AF. Specifically, time-varying covariates (log_2_ UACR and eGFR) were imputed using the 2l.pan method in the mice package, which uses a linear mixed model with homogeneous within-cluster variances (accounting for random intercepts and random slopes). Baseline confounders were imputed using predictive mean matching. Convergence plots and kernel density plots were inspected to assess convergence and imputed values.

To serve as a series of sensitivity analyses, the second approach to handling missing data involved excluding participants with missing baseline data and imputing missing data after baseline using the last observation carried forward (LOCF) method. We refer to this method as ‘complete case analysis/LOCF’.

Subgroup analyses were conducted for the following characteristics: sex, age group, ethnicity, country, BMI, hypertension, diabetes and frailty. Frailty was defined as per the Fried Frailty Score [25]. Description of event numbers and rates in these subgroups used the complete case cohort; interaction terms and adjusted hazard ratios (HRs) were estimated using the truncated marginal structural Cox model with multiple imputation, while event numbers and rates were presented using the complete case cohort. Marginal structural Cox models did not adjust for an irregular heart rate detected at the baseline study visit, due to sample size limitations in some of the subgroups (as irregular heart rate detected at the baseline study visit was uncommon).

All analyses were performed in R version 4.2.2 (R Foundation for Statistical Computing, Vienna, Austria) [26].

## RESULTS

### Characteristics of the study sample

**Figure 1:**
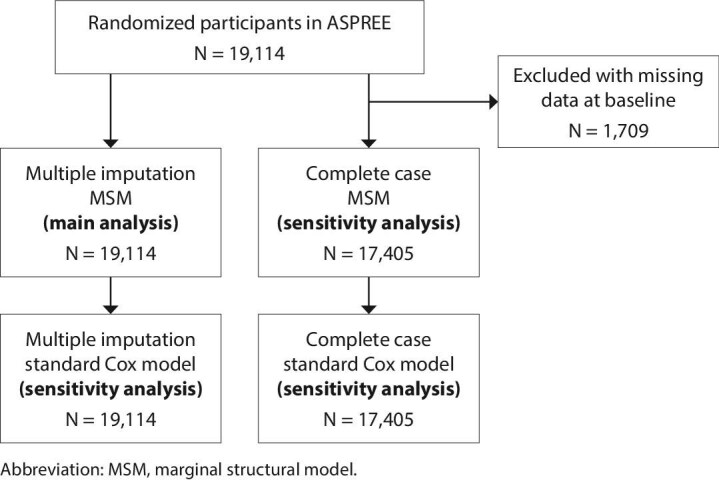
Derivation of the analytic cohorts.

The construction of the analytic cohort is shown in Fig. 1. All 19 114 ASPREE study participants were included in the multiple imputation marginal structural model (MSM) analysis with truncation (primary model), the untruncated multiple imputation MSM and the standard Cox model, which was conducted as a sensitivity analysis. The complete case analysis/LOCF approach (for both the truncated and untruncated MSMs and the standard Cox model) used 17 405 individuals in whom non-missing data at baseline were available.

Characteristics at study baseline of the analytic cohort using multiple imputation are shown in Table 1. Overall, the mean age was 75.1 ± 4.5 years and 56.4% were female. By race/ethnicity, 91.3% were White (85.6% White from Australia and 5.7% White from the USA), 4.7% were African American and 2.6% were Hispanic. Participants with a UACR ≥3 mg/mmol, compared with participants with a UCAR <3 mg/mmol, were relatively more likely to be older, male, a racial or ethnic minority, to have diabetes or hypertension, to have higher SBP or DBP, to have lower HDL and non-HDL cholesterol levels or a lower haemoglobin level, to be a current smoker, to have a higher BMI, to be frail or to use ARBs or ACEIs; they also had lower educational attainment or were more often living at home alone. They also had a slightly lower eGFR level.

**Table 1:** Participant characteristics at baseline.

Characteristics	Total (*N* = 19 114)	Missing UACR (*n* = 983)	UACR 0–<3 mg/mmol (*n* = 16 046)	UACR ≥3 mg/mmol (*n* = 2085)	*P*-value^a^
Age (years), mean (SD)	75.1 (4.5)	75.1 (4.5)	75.0 (4.4)	76.2 (5.2)	<.001
Female, *n* (%)	10 782 (56.4)	560 (57.0)	9145 (57.0)	1077 (51.7)	<.001
Race/ethnicity					<.001
Australian White	16 362 (85.6)	889 (90.4)	13 766 (85.8)	1707 (81.9)	
US White	1088 (5.7)	31 (3.2)	932 (5.8)	125 (6.0)	
African American	901 (4.7)	26 (2.6)	748 (4.7)	127 (6.1)	
Hispanic	488 (2.6)	20 (2.0)	380 (2.4)	88 (4.2)	
Other	275 (1.4)	17 (1.7)	220 (1.4)	38 (1.8)	
Education (years), *n* (%)					.076
<12	8636 (45.2)	461 (46.9)	7198 (44.9)	977 (46.9)	
12–15	5574 (29.2)	285 (29.0)	4677 (29.1)	612 (29.4)	
≥16	4903 (25.7)	236 (24.0)	4171 (26.0)	496 (23.8)	
Living alone, *n* (%)	6251 (32.7)	315 (32.0)	5176 (32.3)	760 (36.5)	<.001
Diabetes, *n* (%)	2045 (10.7)	95 (9.7)	1566 (9.8)	384 (18.4)	<.001
Hypertension, *n* (%)					<.001
None	4919 (25.7)	252 (25.6)	4353 (27.1)	314 (15.1)	
Controlled	4713 (24.7)	260 (26.4)	3937 (24.5)	516 (24.7)	
Uncontrolled	5318 (27.8)	269 (27.4)	4226 (26.3)	823 (39.5)	
Untreated	4164 (21.8)	202 (20.5)	3530 (22.0)	432 (20.7)	
Dyslipidaemia, *n* (%)	12 467 (65.2)	613 (62.4)	10 457 (65.2)	1397 (67.0)	.098
Irregular heart rate, *n* (%)	392 (2.1)	21 (2.1)	320 (2.0%)	51 (2.4)	.171
SBP (mmHg), mean (SD)	139.2 (16.5)	138.9 (16.8)	138.7 (16.3)	143.1 (17.0)	<.001
DBP (mmHg), mean (SD)	77.3 (10.0)	76.7 (9.7)	77.2 (9.9)	77.9 (10.4)	.007
Heart rate (bpm), mean (SD)	70.7 (10.7)	71.3 (10.5)	70.7 (10.7)	70.8 (11.2)	.632
HDL (mmol/l), mean (SD)	1.6 (0.5)	1.6 (0.5)	1.6 (0.5)	1.5 (0.5)	<.001
Non-HDL (mmol/l), mean (SD)	3.7 (0.9)	3.6 (1.0)	3.7 (0.9)	3.6 (1.0)	<.001
Haemoglobin (g/dl), mean (SD)	14.2 (1.2)	14.2 (1.3)	14.2 (1.2)	14.1 (1.3)	.052
Current smoker, *n* (%)	735 (3.8)	32 (3.3)	589 (3.7)	114 (5.5)	<.001
Current alcohol use, *n* (%)	14 642 (76.6)	756 (76.9)	12 356 (77.0)	1530 (73.4)	<.001
BMI (kg/m^2^), mean (SD)	28.1 (4.7)	28.3 (4.7)	28.1 (4.7)	28.3 (5.1)	.061
Frailty, *n* (%)					<.001
Not frail	11 246 (58.8)	577 (58.7)	9639 (60.1)	1030 (49.4)	
Pre-frail	7447 (39.0)	379 (38.6)	6085 (37.9)	983 (47.1)	
Frail	421 (2.2)	27 (2.7)	322 (2.0)	72 (3.5)	
Use of ARBs or ACEIs, *n* (%)	7975 (41.7)	7975 (41.7)	7975 (41.7)	7975 (41.7)	<.001
Polypharmacy, *n* (%)	5088 (26.6)	238 (24.2)	4098 (25.5)	752 (36.1)	<.001
eGFR (ml/min/1.73 m^2^), mean (SD)	76.9 (14.2)	76.5 (14.2)	77.4 (13.8)	73.3 (16.6)	<.001

a *P*-value represents the contrast between participants with a UACR <3 mg/mmol and ≥3 mg/mmol.

Number missing from the total: education, 1; history of irregular heart rate, 3; heart rate, 5; HDL cholesterol, 446; non-HDL cholesterol, 448; haemoglobin 2; BMI, 89; eGFR, 464.

The characteristics of the 17 405 participants are included in the complete case analysis/LOCF compared with the 1709 participants excluded due to missing baseline data (Supplementary Table S1). Participants with missing data were more likely to be White Australian and less likely to have dyslipidaemia or ≥16 years of education.

Median follow-up time for the ASPREE study cohort was 4.7 years (interquartile range 3.6–5.7). UACR values over the course of the study are shown in Table 2. The mean ± 1 SD increased from 2.1 ± 8.2 mg/mmol at baseline to 2.7 ± 12.5 mg/mmol at year 4 to 2.1 ± 3.3 mg/mmol at year 7.

**Table 2:** UACR over the follow-up period for the 19 114 participants included in the main analysis.

Study visit	Participants at risk of AF, *n*	Participants with UACR^a^, *n*	UACR (mg/mmol), mean (SD)
0	19 114	18 131	2.1 (8.2)
1	18 583	13 733	2.2 (9.2)
2	17 974	12 739	2.5 (15.1)
3	15 683	11 427	2.7 (11.9)
4	11 675	7466	2.7 (12.5)
5	7206	4878	2.9 (8.9)
6	2658	1695	2.8 (8.4)
7	104	60	2.1 (3.3)

a Participants providing a urine sample at the yearly visit.

Visit 0 represents the baseline visit of the ASPREE trial.

### Rates of new-onset AF by level of baseline UACR

**Figure 2:**
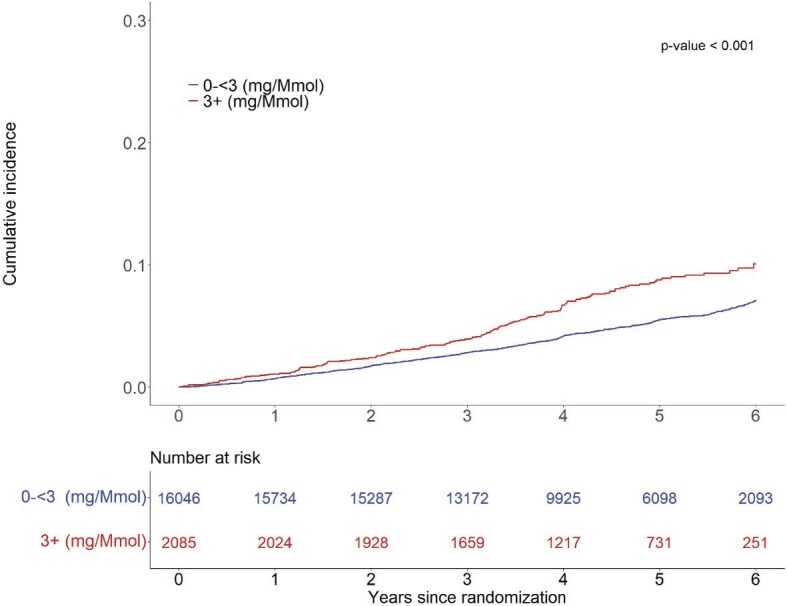
Cumulative incidence of AF, by level of UACR, among participants who have baseline data on UACR (*n* = 18 131).

The rate of developing AF was 1.11/100 person-years (786 events) among participants with UACR <3 mg/mmol at baseline compared with 1.74/100 person-years (155 events) at baseline among participants with UACR ≥3 mg/mmol. The cumulative incidence of AF development is shown in Fig. 2. At 3 years, 2.8% of participants with a UACR <3 mg/mmol developed AF, compared with 3.9% of participants with a UACR ≥3 mg/mmol. At 6 years, 7.1% and 10.1% of participants, respectively, developed AF (*P* < .001).

### Association of UACR with development of AF

The performance of the marginal structural model was examined. Correlation between time-varying confounders and UACR over follow-up before and after weighting is shown in Supplementary Table S2. Satisfactory balance for the time-varying confounders was achieved in the truncated MSM, although acute myocardial infarction and hospitalization for heart failure at year 7 (only) was unbalanced in the untruncated weights.

The risk of AF development is shown in Table 3, in which adjusted HRs (aHRs) are shown for a doubling of UACR. The aHR for AF in the marginal structural model with weight truncation was 1.160 (95% CI 1.114–1.207) with multiple imputation and 1.145 (95% CI 1.100–1.191) in the complete case analysis/LOCF. Respective results were virtually identical for the model with untruncated weights for both multiple imputation and complete case analysis/LOCF. The aHRs were marginally lower for the Cox model: 1.146 (95% CI 1.102–1.192) for the multiple imputation model and 1.134 (95% CI 1.091–1.179) for the complete case analysis/LOCF model. All *P*-values were <.001.

**Table 3:** Association between UACR (log_2_) and development of AF.

	Multiple imputation^a^ (*n* = 19 114)		Complete case analysis/LOCF (*n* = 17 405)	
Modelling approach	aHR (95% CI)^b^	*P*-value	aHR (95% CI)^b^	*P*-value
Marginal structural Cox model, truncated^c^	1.160 (1.114–1.207)	<.001	1.145 (1.100, 1.191)	<.001
Marginal structural Cox model, untruncated^c^	1.160 (1.115–1.208)	<.001	1.145 (1.100, 1.192)	<.001
Standard Cox model	1.146 (1.102–1.192)	<.001	1.134 (1.091, 1.179)	<.001

a Multiple imputation models included year, treatment arm, age, sex, race/ethnicity, education level, living situation, diabetes, hypertension, dyslipidaemia, history of irregular heart rate, heart rate, HDL cholesterol, non-HDL cholesterol, haemoglobin, smoking status, alcohol use, BMI, frailty category, use of ARBs or ACEIs, polypharmacy, eGFR, myocardial infarction, hospitalization for heart failure, AF status and cumulative hazard of AF.

b Adjusted for fixed characteristics: treatment arm, age, sex, race/ethnicity, education level, living situation, diabetes, hypertension, dyslipidaemia, history of irregular heart rate, heart rate, HDL cholesterol, non-HDL cholesterol, haemoglobin, smoking status, alcohol use, BMI, frailty category, use of ARBs or ACEIs, polypharmacy. Note that time-varying eGFR and myocardial infarction/hospitalization for heart failure were accounted for by the inverse probability weights.

c Marginal structural model: denominator model included cubic spline year, treatment arm, age, sex, race/ethnicity, education level, living situation, diabetes, hypertension, dyslipidaemia, history of irregular heart rate, heart rate, HDL cholesterol, non-HDL cholesterol, haemoglobin, smoking status, alcohol use, BMI, frailty category, use of ARBs or ACEIs, polypharmacy, eGFR, myocardial infarction and hospitalization for heart failure. Numerator included the same except for eGFR, myocardial infarction and hospitalization for heart failure.

The subgroup analysis, illustrated in Supplementary Fig. S1, showed that the results were generally similar across the strata of each covariate except diabetes status. An interaction between diabetes and UACR (*P* = .010) was found such that UACR increased the risk of AF in participants without diabetes [aHR 1.183 (95% CI 1.134–1.235)] but not for participants with diabetes [aHR 1.028 (95% CI 0.914–1.156)].

## DISCUSSION

In this *post hoc* analysis of ASPREE participants, we examined the association between albuminuria and incident AF in a large group of older individuals with no prior major cardiovascular events. We found that a doubling of UACR was associated with an approximate 16% increase in the risk of AF, independent of a wide range of demographic and clinical factors including eGFR. The results were consistent across a series of modelling approaches, including the approach to missing data. At the end of 6 years, about 1 in 10 individuals with a UACR ≥3 mg/mmol developed AF, compared with about 1 in 15 individuals with a UACR <3 mg/mmol.

A recent systematic review and meta-analysis identified studies that explored the association of albuminuria with incident AF, independent of the effects of eGFR [4]. Four published reports are most relevant to our study, three of which drew from community-based longitudinal cohorts including ARIC (Atherosclerosis Risk in Communities) [3], PREVEND (Prevention of Renal and Vascular ENd-stage Disease) [5], JHS (Jackson Heart Study) [6] and MESA (Multi-Ethnic Study of Atherosclerosis) [6] and one that used a large, comprehensive health system database from Ontario [9]. In each of the studies, higher levels of (baseline) albuminuria were associated with an increased risk of development of AF. Our study differs from these in being larger and consisting of generally healthy older individuals without established cardiovascular disease in whom repeated (rather than a single baseline) measures of UACR were available. The only report in which the results were expressed akin to ours (i.e. as an annual risk of AF associated with a doubling of UACR, was the report that meta-analysed the JHS and MESA cohorts [6]; the models generated in that report yielded AF risk estimates of ≈1.12, which were similar to our own.

The association of albuminuria with AF was similar by strata of age, sex, race/ethnicity, study country, hypertension, BMI and frailty—indeed, all analysed subgroups with the exception of diabetes. We found an interaction between diabetes status and UACR for the development of AF such that UACR was not associated with an increased risk of AF in participants with diabetes. This was unexpected; if anything, one might hypothesize that albuminuria among people with diabetes would confer an especially elevated risk of poor outcomes, such as the development of AF. Reasons for this are unclear, but it may be because individuals with diabetes are at undue risk of AF due to unique causal pathways attributable to their disease [27, 28]. For example, a recent study reported that, even among diabetic individuals with good glycaemic control and normal levels of albumin excretion, the risk of AF was elevated [29]. It is therefore possible that processes associated with diabetes, such as transformation of epicardial adipose tissue causing myocardial fibrosis or abnormalities of autonomic dysfunction [30, 31], confer an especially high risk of AF that subsumes any (more modest) contribution of albuminuria to AF risk. Alternatively, individuals with more severe diabetes—and those most likely to be at greatest risk of the development of AF—were excluded, by design, from ASPREE. A fuller understanding of this phenomenon in our study population would require highly granular data on the level of glycaemic control that are not available in ASPREE.

To extend the current understanding of the risk of AF associated with albuminuria, we leveraged the strengths of ASPREE and incorporated design and analytical approaches that have not, to our knowledge, previously been applied to the study of albuminuria and AF. First, rather than ascribe all downstream risk of AF to a single (baseline) measurement of urinary albumin excretion, as in most previous studies [4], we utilized repeated UACR measurements that were available annually for most participants. By leveraging repeated UACR measurements, the association between time-varying UACR and the outcome of interest, AF, could be rigorously quantified. Second, we utilized a marginal structural model designed to control for the effects of confounders that change over time [32]. In the case of the present study, we reasoned that albuminuria could well be a risk factor for key intercurrent events, such as acute myocardial infarction or hospitalization for (decompensated) heart failure—events that themselves may increase albuminuria and are risk factors for new-onset AF in their own right. Finally, to reduce the risk that data available were unnecessarily excluded or participants censored prematurely, we imputed data; the results from the sensitivity generated from the complete case analysis set were similar to those of the main analysis using imputation, demonstrating robustness in our findings. An additional strength of this analysis is that we were able to investigate our research question in a population relatively unconfounded by a high comorbidity burden, which is important because albuminuria, CKD and other key comorbidities often coexist [33].

The mechanism by which albuminuria may be associated with AF is uncertain. Albuminuria, particularly at low levels, is likely a marker of vascular heath, reflecting processes like endothelial (dys)function, inflammation and sympathetic activation, all of which may, in turn, predispose to the development of AF. As such, the potential impact of our findings on therapeutic interventions should be considered. Inhibition of the renin–angiotensin–aldosterone system (RAAS) with, for example, ACEIs or ARBs, reliably reduces albuminuria and have long been a mainstay of treatment in people with albuminuria [34, 35]. Whether these [36] or other agents [37] designed to reduce albuminuria can reliably decrease the risk of new-onset AF depends on whether the reduction of albuminuria reflects amelioration of the underlying processes for which albuminuria is a marker—endothelial dysfunction, inflammation, sympathetic activation, activation of the clotting cascade and likely others [7–9]. If RAAS inhibition did indeed have a salutary effect on these underlying pathophysiologic processes, there would still be a question of whether widespread prescription of RAAS inhibitors to people with no other indication for such drugs would be an appropriate trade-off. Given the well-known risks of drug intolerance and adverse events associated with RAAS inhibitors, not to mention the associated costs, no evidence exists, at present, for this approach.

Although the ASPREE trial provided a unique opportunity to study the association of albuminuria with AF in healthy older adults, there are also limitations to our study. The findings of our study apply only to generally healthy older individuals. The vast majority of ASPREE participants were White, meaning that the findings may not be applicable to populations with more racial and ethnic diversity. AF was not a primary or secondary outcome in ASPREE, and electrocardiograms were not conducted as part of the study protocol, thus AF was likely underdetected, although this may not have varied by level of albuminuria. Paroxysmal AF that did not result in a diagnosis of AF in the medical record or new use of anticoagulants was not included. Although we controlled for a large number of covariates that could be associated with the development of AF, the ASPREE clinical trial was unable to account for all conditions that could, in theory, contribute to the development of AF, such as thyroid disease or asymptomatic chronic ischaemic heart disease. Likewise, we did not have access to data that would indicate the presence of valvular heart disease (which is an aetiology of AF), as it was beyond the scope of the trial to ascertain echocardiographic data on ASPREE participants; it is possible that a small percentage of AF cases were attributable to valvular heart disease. In addition, we did not have access to markers of inflammation, such as C-reactive protein, which has been associated with the risk of AF.

In summary, in healthy older individuals who had generally low levels of urinary albumin excretion, doubling of UACR was associated with an approximate 16% increase in the risk of new-onset AF. Future studies are needed to test whether new biomarkers predict the risk of AF or if a reduction in albuminuria using RAAS inhibitors or novel therapeutics, such as glucagon-like peptide-1 agonists, may reduce AF risk.

## Supplementary Material

sfaf119_Supplemental_File

## Data Availability

The data from the ASPREE randomized trial cohort supporting the findings of this study are available for qualified researchers upon application at https://ams.aspree.org/public/request-data/access-aspree-data/.
