# Supplementary material for: Association of albuminuria with incident atrial fibrillation in healthy older adults
Source: Clin Kidney J. 2025 Apr 17;18(6):sfaf119. doi: 10.1093/ckj/sfaf119 (PMC12128063; doi:10.1093/ckj/sfaf119)
Supplement: sfaf119_Supplemental_File [file sfaf119_supplemental_file.docx]

**Supplemental Material** for *Association between albuminuria and incident atrial fibrillation in healthy older adults*

**Supplemental Table 1.** Participant baseline characteristics for those with missing data at baseline (missing cases) and those without missing data at baseline (complete cases).

**Supplemental Table 2.** Correlation of time-varying urine albumin-to-creatinine ratio (log base 2) with time-varying eGFR and myocardial infarction/hospitalization for heart failure.

**Supplemental Figure 1.** Subgroup analysis of the association of urine albumin-to-creatinine ratio (log base 2) with development of atrial fibrillation.

**Supplemental Table 1.** Participant baseline characteristics for those with missing data at baseline (missing cases) and those without missing data at baseline (complete cases).

| **Characteristic** | **Complete cases**^a^  ***(n* = 17,405)** | **Missing cases**^b^  **(*n* = 1709)** | **p-value** |
| --- | --- | --- | --- |
| **Age***, yrs* | 75.1 (4.5) | 75.2 (4.5) | 0.514 |
| **Female sex** | 9834 (56.5%) | 948 (55.5%) | 0.413 |
| **Race/Ethnicity** |  |  | < 0.001 |
| Australian White | 14785 (84.9%) | 1577 (92.3%) |  |
| US White | 1052 (6.0%) | 36 (2.1%) |  |
| African American | 868 (5.0%) | 33 (1.9%) |  |
| Hispanic | 455 (2.6%) | 33 (1.9%) |  |
| Other | 245 (1.4%) | 30 (1.8%) |  |
| **Education**, *yrs* |  |  | 0.015 |
| <12 | 7810 (44.9%) | 826 (48.4%) |  |
| 12-15 | 5092 (29.3%) | 482 (28.2%) |  |
| ≥16 | 4503 (25.9%) | 400 (23.4%) |  |
| **Living alone** | 5694 (32.7%) | 557 (32.6%) | 0.918 |
| **Diabetes** | 1869 (10.7%) | 176 (10.3%) | 0.575 |
| **Hypertension** |  |  | 0.993 |
| No hyp | 4480 (25.7%) | 439 (25.7%) |  |
| Controlled hyp | 4288 (24.6%) | 425 (24.9%) |  |
| Uncontrolled hyp | 4847 (27.8%) | 471 (27.6%) |  |
| Untreated hyp | 3790 (21.8%) | 374 (21.9%) |  |
| **Dyslipidemia** | 11408 (65.5%) | 1059 (62.0%) | 0.003 |
| **Irregular heart rate** | 350 (2.0%) | 42 (2.5%) | 0.210 |
| **SBP,** mmHg | 139.2 (16.5) | 139.2 (16.7) | 0.963 |
| **DBP,** mmHg | 77.3 (10.0) | 76.9 (9.9) | 0.106 |
| **Heart rate,** bpm | 70.7 (10.8) | 71.0 (10.5) | 0.275 |
| **HDL,** mmol/L | 1.6 (0.5) | 1.6 (0.5) | 0.901 |
| **Non-HDL**, mmol/L | 3.7 (0.9) | 3.7 (1.0) | 0.753 |
| **Hemoglobin,** g/dL | 14.2 (1.2) | 14.2 (1.3) | 0.055 |
| **Current smoker** | 677 (3.9%) | 58 (3.4%) | 0.596 |
| **Current alcohol use** | 13327 (76.6%) | 1315 (76.9%) | 0.654 |
| **BMI,** kg/m^2^ | 28.1 (4.7) | 28.2 (4.8) | 0.166 |
| **Frailty** |  |  | 0.386 |
| Not frail | 10221 (58.7%) | 1025 (60.0%) |  |
| Pre-frail | 6805 (39.1%) | 642 (37.6%) |  |
| Frail | 379 (2.2%) | 42 (2.5%) |  |
| **Use of ARBs or ACEIs** | 7244 (41.6%) | 731 (42.8%) | 0.356 |
| **Polypharmacy** | 4656 (26.8%) | 432 (25.3%) | 0.189 |
| **eGFR**^c^ | 76.9 (14.2) | 76.5 (14.3) | 0.317 |

Continuous values reported as mean (± 1 standard deviation); other values reported as a percentage.

^a^Complete cases are participants with no missing data at baseline.

^b^Missing cases are participants with missing data at baseline in at least one variable included in the analysis: age, sex, race/ethnicity, education level, living situation, diabetes, hypertension, dyslipidemia, history of irregular heart rate, heart rate, HDL cholesterol, non-HDL cholesterol, hemoglobin level, smoking status, alcohol use, BMI, frailty category, and polypharmacy.

^c^eGFR measured as mL/min/1.73m^2^; mean score shown.

Number missing from the total: Education, 1; history of irregular heart rate, 3; heart rate, 5; HDL, 446; non-HDL, 448; hemoglobin 2; BMI, 89; eGFR, 464.

Abbreviations: UACR, urine albumin-creatinine ratio; hyp, hypertension; SBP, systolic blood pressure; DBP, diastolic blood pressure; BMI, body mass index; eGFR, estimated glomerular filtration rate.

**Supplemental Table 2.** Correlation of longitudinal urine albumin-to-creatinine ratio (log base 2) with longitudinal eGFR and myocardial infarction/hospitalization for heart failure.

| **Year** | **Characteristic** | **Pearson** **correlation coefficient with Log base 2 UACR** | | |
| --- | --- | --- | --- | --- |
|  |  | **Unweighted** | **Weighted, truncated** | **Weighted, untruncated** |
| **0** | **eGFR** | -0.067 | -0.027 | -0.027 |
| **1** | **eGFR** | -0.087 | -0.033 | -0.033 |
|  | **MI/HHF** | 0.024 | 0.004 | 0.004 |
| **2** | **eGFR** | -0.095 | -0.024 | -0.023 |
|  | **MI/HHF** | 0.017 | -0.011 | -0.013 |
| **3** | **eGFR** | -0.105 | -0.015 | -0.012 |
|  | **MI/HHF** | 0.025 | -0.013 | -0.018 |
| **4** | **eGFR** | -0.109 | -0.008 | -0.003 |
|  | **MI/HHF** | 0.024 | -0.019 | -0.031 |
| **5** | **eGFR** | -0.101 | 0.015 | 0.024 |
|  | **MI/HHF** | 0.023 | -0.030 | -0.054 |
| **6** | **eGFR** | -0.117 | 0.005 | 0.019 |
|  | **MI/HHF** | 0.038 | -0.009 | -0.037 |
| **7** | **eGFR** | -0.130 | -0.008 | 0.031 |
|  | **MI/HHF** | -0.039 | -0.077 | -0.172 |

Truncated models were truncated at the 0.1^th^ and 99.9^th^ percentiles.

Year 0 represents the baseline.

Weighted represents the average correlation across imputed datasets.

Abbreviations: UACR, urine albumin-to-creatinine ratio; eGFR, estimated glomerular filtration rate; MI, myocardial infarction; HHF, hospitalization for heart failure.

**Supplemental Figure 1.** Subgroup analysis of the association of urine albumin-to-creatinine ratio (log base 2) with development of atrial fibrillation.


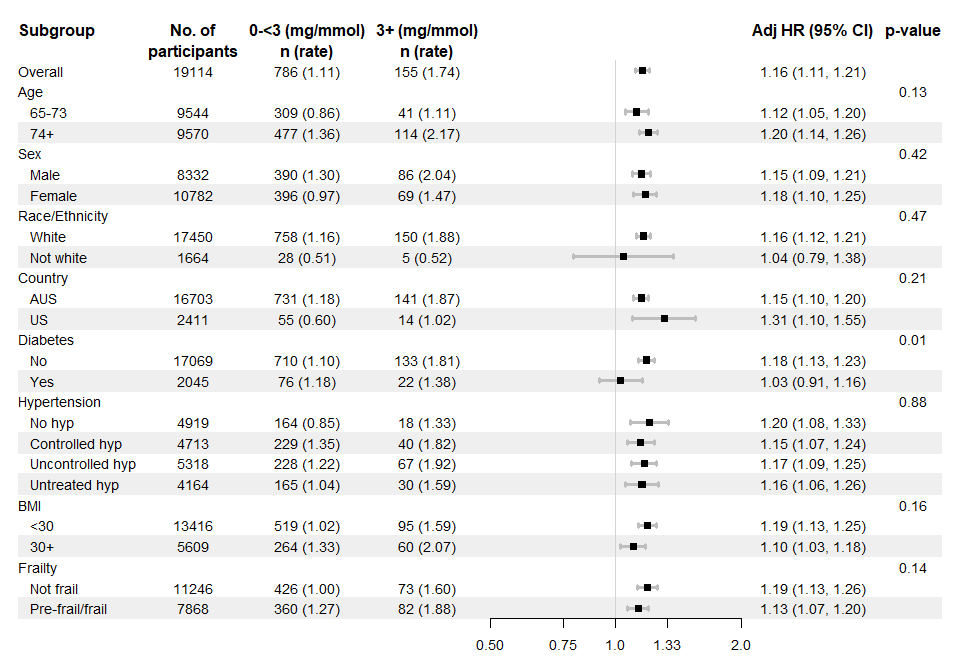


Abbreviations: ADJ HR, adjusted hazard ratio; CI, confidence interval; hyp; hypertension; BMI, body mass index.
